# Endometrioid Adenocarcinoma of Caecum Causing Intussusception

**DOI:** 10.1155/2013/714126

**Published:** 2013-04-28

**Authors:** Rashmi Verma, Sally Osborn, Kieran Horgan

**Affiliations:** ^1^Department of Breast Surgery, St James's Hospital, Beckett Street, Leeds, West Yorkshire LS97TF, UK; ^2^Department of Pathology, St James's Hospital, Beckett Street, Leeds, West Yorkshire LS97TF, UK

## Abstract

Malignant transformation of endometriosis is rare and is usually seen in ovarian endometriosis. The colon and rectum are the most common sites for extragonadal endometriosis, and although serosal involvement is commonly seen, mucosal involvement is rare. Malignant transformation of endometriosis is a rare but a well-known complication of endometriosis. We report an unusual presentation of endometrioid adenocarcinoma with lymph node metastasis, arising from endometriosis in the caecal wall and causing ileocaecal intussusception. The patient presented with sudden onset of abdominal pain with features suggestive of acute appendicitis. Diagnostic laparoscopy revealed an ileocaecal intussusception. Conversion to open surgery confirmed a caecal mass causing ileocaecal intussusception, and a radical right hemicolectomy was performed. Histology revealed endometrioid adenocarcinoma arising in a focus of endometriosis in the muscularis propria and involving the mucosa, with one regional metastatic lymph node.

## 1. Introduction

Endometriosis is defined as the presence of endometrial tissue in extrauterine sites and can be found in the ovaries, fallopian tubes, broad ligaments, cervix, pouch of Douglas, small and large intestines, recto vaginal septum, and the appendix. The colon and rectum are the most common sites for extragonadal endometriosis, and although serosal involvement is commonly seen, mucosal involvement is rare. Malignant transformation of endometriosis is rare and is usually found in ovarian endometriosis. The most common extrapelvic site for malignant transformation is the gastro-intestinal tract, and the majority of cases are reported in the sigmoid colon or rectum. Endometrioid adenocarcinoma is the most common histological type of malignant tumour to arise in endometriosis [1]. A literature search revealed only one case of endometrioid carcinoma of the caecum and terminal ileum [2] which was a mixed adenosquamous carcinoma. 

Although intussusception is primarily seen in children, adult intussusception is a rare cause of abdominal pain and forms 5% of all intussusceptions [3]. In contrast to intussusception in children, it is always associated with an obvious pathology [4]. We report an unusual presentation of endometrioid adenocarcinoma of the caecal pole causing intussusception.

## 2. Case Report

A 49-year-old lady presented with sudden onset abdominal pain. It commenced as a central abdominal pain and later shifted to the right iliac fossa. There was associated nausea but no vomiting, fever, or change in bowel habit. Clinical examination revealed tenderness in the right iliac fossa. Blood tests at admission showed raised inflammatory markers (WCC: 15.9, CRP: 61) and no other abnormality.

In her past medical history, she was diagnosed with pelvic endometriosis in 1999 and underwent hysterectomy and bilateral salpingo-oophorectomy in 2000. She was on oestrogen replacement therapy since then. Clinical symptoms were suggestive of acute appendicitis, and she underwent an emergency diagnostic laparoscopy. At laparoscopy, an ileocaecal mass suspicious of malignancy was found and the procedure was converted to open surgery. A caecal mass causing ileocaecal intussusception was identified and a radical right hemicolectomy was performed. She made a good postoperative recovery. Staging CT scan performed postoperatively did not reveal any residual disease or any evidence of metastasis.

*Histopathology*. Macroscopically, there was an intussusception of the caecal pole and ileum (intussusceptum) into the caecum/colon. The intussuscepted mass measured 55 × 45 × 45 mm and the mucosa of the intussuscepiens was oedematous but smooth with no obvious ulceration or necrosis. 

**Figure 1:**
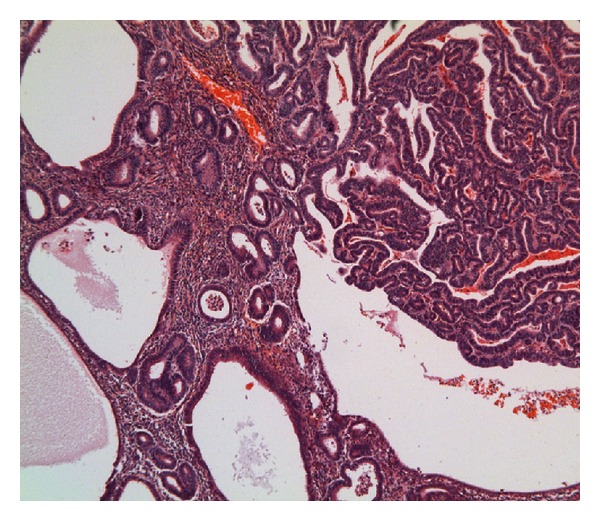
Colon with endometrioid adenocarcinoma (right side) adjacent to endometriosis (left).

**Figure 2:**
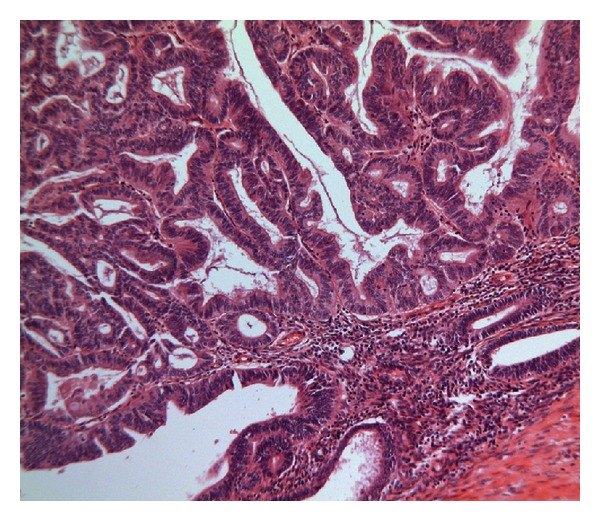
Endometrioid adenocarcinoma (left) adjacent to endometriosis and muscularis (bottom right).

**Figure 3:**
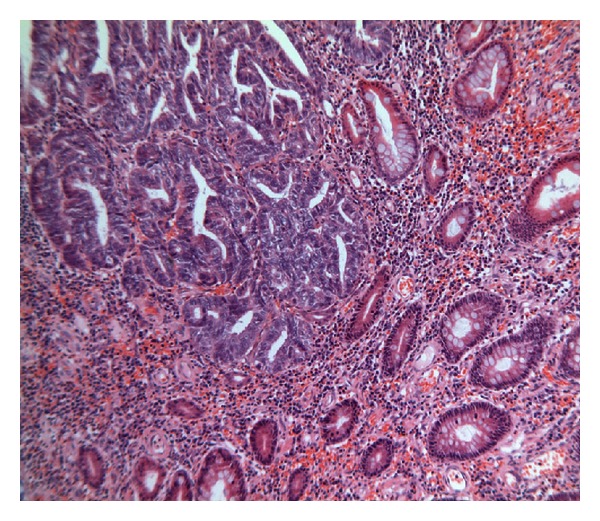
Endometrioid adenocarcinoma (top left) next to normal bowel mucosa (bottom right), that is, no dysplasia of bowel mucosa.

**Figure 4:**
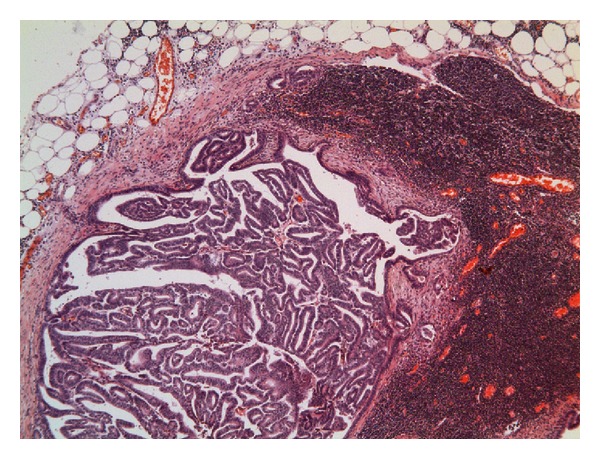
Metastatic endometrioid adenocarcinoma in a lymph node.

Microscopic examination revealed that the head of the intussusceptum contained a well-differentiated grade 1 endometrioid adenocarcinoma arising in a focus of endometriosis centered in the muscularis propria of the intussuscepted caecum (Figures 1 and 2). Owing to the extrauterine origin of the tumour, a TNM/FIGO stage could not be assigned to this tumour. Tumour was however confined to the bowel wall (predominantly muscularis propria with focal areas of involvement of the overlying mucosa) (Figure 3). Small foci of endometriosis were seen scattered transmurally in the mucosa, muscularis propria, and subserosa in the caecum around the main focus. Small, discrete, scattered satellite foci of endometrioid adenocarcinoma were seen in the mucosa of the caecum close to and overlying the main tumour in the muscularis propria. Histological contiguity between these foci and the main tumour could not be demonstrated. These foci were thought to represent angiolymphoid spread from the main tumour or adenocarcinoma arising in mucosal endometriotic foci as part of a more widespread field change. There was no evidence of serosal involvement by the tumour. Metastatic adenocarcinoma was present in 1 out of 29 lymph nodes (Figure 4). Occasional nonmalignant endometriotic foci were seen in the perinodal fat. 

## 3. Discussion

The gastrointestinal tract is the most common site of extrapelvic endometriosis. Within the gastrointestinal tract, the rectum and sigmoid colon are the most commonly involved sites of extrapelvic endometriosis. The pathogenesis of endometriosis remains uncertain. Retrograde menstruation and endometrial metaplasia with displacement of endometrial tissue are the most favoured explanations. However, it has been proposed that haematogenous or lymphatic dissemination of endometrial cells may also play a role [5].

Malignant transformation of endometriosis in gonadal and extragonadal tissue is well documented. In 1925, Sampson reported 7 cases of malignant transformation of gonadal and extra-gonadal endometriosis [6]. Although the precise incidence of malignant transformation of endometriosis is unknown, a 0.7–1% incidence has been reported in the literature [5]. Seventy five percent of cases of malignant transformation occur in the ovary, and the remainder are usually in the pelvis, in extragonadal sites such as the pelvic peritoneum, the recto vaginal septum, sigmoid colon, rectum, and vagina [7]. A literature search revealed only one case involving the caecum and terminal ileum which was an adenosquamous carcinoma.

Intramural intestinal endometrioid adenocarcinoma is relatively rare. Only 14 cases have been described in the literature [7]. Of these, 5 cases had mucosal involvement. Mucosal involvement is not typically seen in cancers arising from endometriosis in the intestinal tract and is in fact one of the ways in which it can be distinguished from a primary intestinal adenocarcinoma [8]. Lymph node metastasis from intestinal endometrioid adenocarcinoma is also quite rare. Only seven cases, including ours, are documented in the literature [1, 2, 5, 7–9]. 

All previously reported cases have stressed the importance of immunohistochemical staining for CK7, CK20, and estrogen receptor (ER) to diagnose endometrioid adenocarcinoma and to distinguish it from colonic adenocarcinoma. However, definite diagnosis of endometrioid adenocarcinoma does not always require immunostaining. In our case, the diagnosis was possible on histological characteristics alone.

Hyperestrogenism has been implicated as a risk factor for malignant transformation of gonadal and extra-gonadal endometriosis. In vitro, estrogens show a proliferative effect on ovarian carcinoma cell lines with progesterone, and antiestrogens found to have an antiproliferative effect. Estrogen-only hormonal replacement therapy was found to be associated with malignant transformation of endometriosis [1, 10]. In a review of 205 cases including gonadal and extra gonadal sites by Heaps et al., 14 cases occurred on a background of oestrogen stimulation [11]. Debus and Schuhmacher showed a similar association in 2001, and therefore they recommended treating patients with endometriosis with combination replacement therapy [12]. Our patient was taking oestrogen replacement therapy for 11 years.

Treatment of advanced endometrioid cancer that has arisen in endometriosis is difficult. There is no established treatment protocol for colonic endometrioid adenocarcinomas, and the use of adjuvant chemotherapy is controversial. The use of platinum drugs, anthracyclines, and paclitaxel has been described in the literature [10]. Our patient was treated with Paclitaxel and Carboplatin. The value of chemotherapy to prevent relapse following complete surgical resection is uncertain and requires further investigations.
